# MRI appearances of hepatic epithelioid hemangioendothelioma: a retrospective study of 57 patients

**DOI:** 10.1186/s13244-022-01213-8

**Published:** 2022-04-05

**Authors:** Xiaolei Liu, Hongwei Yu, Zihuan Zhang, Shuang Si, Jia Huang, Haidong Tan, Feng Teng, Zhiying Yang

**Affiliations:** 1 Department of Hepatobiliary Surgery, China-Japan Friendship Hospital, 2 Yinghua Dongjie, Hepingli, Beijing, 100029 China; 2 Department of Radiology, China-Japan Friendship Hospital, Beijing, China; 3 Department of Radiation Oncology, China-Japan Friendship Hospital, 2 Yinghua Dongjie, Hepingli, Beijing, 100029 China

**Keywords:** Hepatic epithelioid hemangioendothelioma, HEH, MRI, Hepatic tumors, Rare liver tumors

## Abstract

**Background:**

Hepatic epithelioid hemangioendothelioma (HEH) is extremely rare and the MRI features have never been investigated in a large group of patients.

**Methods:**

A retrospective study was designed to review the MRI images of HEH patients. Two radiologists separately evaluated signal intensity (SI) on unenhanced imaging, morphological features, contrast-enhancement pattern at dynamic study. The MRI features were compared between patients with HEH and hepatic metastatic tumor (HMT).

**Results:**

Fifty-seven HEH patients were included in this study and a total of 412 lesions were evaluated. On per-lesion analysis, the rate of coalescent lesion and subcapsular lesion were 18.2% and 39.8%, respectively. Capsular retraction and lollipop sign were observed in 47 lesions (11.4%) and 60 lesions (14.6%), respectively. Large lesions (> 5 cm) had the highest rate of coalescent lesion, subcapsular lesion, capsular retraction and lollipop sign. Target sign appeared in 196 lesions (47.6%) on T2 weighted (T2W) and 146 lesions (35.4%) on portal phase. Medium lesions (2–5 cm) had the highest rate of target sign on both T2W (72.9%) and portal phase (55.2%). On per-patient analysis, compare with HEH patients, HMT patients seldom had the appearance of lollipop sign (66.7% versus 6.4%, *p* < 0.01), capsular retraction (59.6% versus 3.2%, *p* < 0.01) and target appearance on both T2Wand portal phase (64.9% versus 12.7%, *p* < 0.01).

**Conclusion:**

MRI features of HEH correlated with the lesion size. Capsular retraction, lollipop sign and coexistence of target sign on both T2W and portal phase were relatively specific MRI features of HEH, which could be helpful in suggesting the diagnosis.

## Key Points

- Hepatic metastatic tumors (HMT) have high rates of subcapsular lesion, coalescent lesion and target sign on one sequence, which makes them less specific MRI features for hepatic epithelioid hemangioendothelioma (HEH) patients.
- Lollipop sign, capsular retraction and target sign on both T2W and portal phase seldom occur in HMT patients, and they are more specific MRI features for HEH patients.
- The MRI features of HEH correlate with the lesion size. Large lesions (> 5 cm) have the highest rate of capsular retraction and lollipop sign, while target sign is more likely to be found in medium lesions (2–5 cm).

## Introduction

Hepatic epithelioid hemangioendothelioma (HEH) is an extremely rare liver tumor [1]. It originates from the vascular endothelium, and the biological behavior of the tumor is deemed between angiosarcoma and hemangioma [2]. For most patients, the tumors progress slowly and may regress spontaneously without any treatment [3]. The tumors are more likely detected accidentally by ultrasonography or computed tomography (CT) examination with no clinical symptom [4]. Usually, multiple intrahepatic lesions occur at the time of detection, and only a few patients have singular lesion [5]. Meanwhile, extrahepatic lesions located in lung, spleen and bone may be found simultaneously [4, 6]. Due to the rarity of the disease, HEH is often misdiagnosed as cholangiocarcinoma or metastatic tumor on imaging.

Although final diagnosis relies on pathological examination, radiological features of HEH are still valuable for differential diagnosis. Several studies have reported the magnetic resonance imaging (MRI) features of HEH, including subcapsular lesions, coalescent lesions, capsular retraction, target sign and lollipop sign [7–10]. However, the number of included patients in these studies was limited, which made the results less consistent. According to our experience, the reported radiological features of HEH may not be all manifested on one patient, and the probability of characteristic MRI appearance may differ among lesions of different sizes. Meanwhile, no study has ever compared the MRI features of HEH with hepatic metastatic tumors (HMT). From 2014, our team has been investigating HEH and a total of 72 patients are under regular follow-up right now. This study was aimed to provide a comprehensive view of MRI features in a large group of HEH patients, analyze the difference of radiological features among lesions of different sizes and explore the most valuable and convenient MRI features to differentiate HEH from HMT.

## Patients and methods

### Patients

From March 2014 to September 2021, 72 histologically diagnosed HEH patients (including 60 patients by liver biopsy and 12 patients by surgery) were followed up regularly by our team and all their clinical and radiological data were collected. A retrospective study was designed to review the MRI images of all HEH patients. All of the 72 HEH patients’ radiological database was reviewed and patients with an abdominal contrast-enhanced MRI examination performed prior to any kind of treatment (surgery, target therapy, interferon-a 2b, radiofrequency ablation or chemotherapy) were included in this study. To compare the MRI features between HEH and HMT patients, histologically diagnosed HMT patients with contrast-enhanced MRI examination from September 2020 to September 2021 in our center were also reviewed and only patients with pre-treatment MRI images were included in this study. Institutional Review Board exempted the study from formal approval due to its retrospective and noninvasive nature. Since patient’s privacy was maintained and no impact was implemented on patient’s care, patient consent was not required for this retrospective study.

### MRI Imaging

To minimize the bias caused by technical disparity, the minimal technical requirements of MRI for both HEH and HMT patients were as follows: using a 3.0 T magnet equipped with parallel imaging, a gradient strength > 35 mT/m, and torso-array coil (with a minimum of an eight-receive channel for signal reception). For patients with multiple pre-treatment MRI examinations, the MRI scan with optimal image quality were included. Imaging protocol should include: unenhanced T1W imaging and dynamic contrast enhanced (using either gadobenate dimeglumine or gadolinium ethoxybenzyl diethylenetriamine pentaacetic acid) study using gradient recall echo (GRE) 2D T1-weighted (W) in-phase, out-of-phase and/or GRE 3D T1W sequence with spectral fat saturation acquiring in the breath-hold arterial phase, portal phase and equilibrium phase; T2W sequences (turbo spin echo [TSE] T2W respiratory triggered with spectral fat saturation or single-shot-TSE T2W with breath-hold acquisition); and diffusion-weighted imaging (DWI).

### Image analysis

Two skilled radiologists with specialty in abdominal imaging and liver MRI separately evaluated the MRI images. Both of the reviewers were blind to the histology results. Reviewers separately evaluated signal intensity (SI) on unenhanced imaging, morphological features, contrast-enhancement pattern at dynamic study (ring-like: peripheral enhancement around the lesion, core, target-like, or heterogeneous enhancement) and SI on DWI (evaluated on high b-values images). HEH morphological features that reviewers evaluated and recorded separately included the following items: number of lesions, lesion type (nodular lesion; coalescent lesion: lesion seemed to be formed by overlapping lesions; diffuse lesion: lesions had no clear margins), size, location, presence of subcapsular lesion, capsular retraction (adjacent liver surface was retracted toward the lesion), “target sign” (two or multiple concentric layered “target-like” appearance on any sequence) and “lollipop sign” (the hypodense well defined tumor on enhanced images as the “candy” and an obstructed or occluded vein as the “stick”). Each lesion was evaluated, respectively, and the ten largest lesions were evaluated for patients with more than 10 lesions. Descriptive analysis of each finding was performed on both per-lesion and per-patient basis in this study. In per-lesion analysis, coalescent or diffuse lesion was regarded as one lesion to be counted and evaluated. When discordant opinions occurred between the two reviewers, a joint review was held to reach a consensus.

### Statistical analysis

The measurement data were represented as means ± SDs. Between-group comparisons were performed using the one-way ANOVA. Count data were represented as frequencies or rates, and the chi-square tests were performed. Differences with *p* < 0.05 were considered significant. The data were analyzed using SPSS 24.0.

## Results

### General information

From March 2014 to September 2021, a total of 72 HEH patients with detailed clinical and radiological data were followed up regularly. Fifteen patients were excluded, since 9 patients had no pre-treatment contrast enhanced MRI and 4 patients’ MRI didn’t meet the technical requirements. Finally, 57 HEH patients (male 32, female 25; median age: 35 years old, range: 15–65 years old) were included in this study. Twenty-nine (50.9%) patients had extrahepatic metastasis, including lung (24 patients, 42.1%), bone (2 patients 3.5%), lung + bone (1 patient, 1.8%), lung + spleen (1 patient, 1.8%), and lung + peritoneum (1 patient, 1.8%). Five patients (8.8%) had singular intrahepatic lesion, 24 patients (42.1%) had more than 10 intrahepatic lesions and 28 patients (49.1%) had 2–10 intrahepatic lesions.

### MRI findings of HEH on per-lesion analysis

**Fig. 1:**
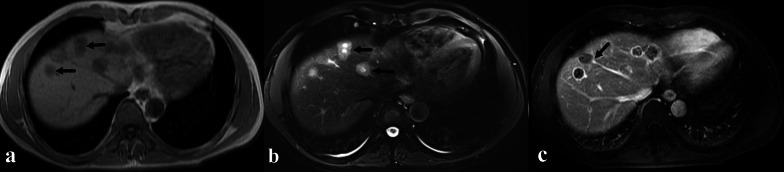
A 42-year-old male HEH patient. **a** Coalescent lesion with target sign on T1-weighted 3D GRE image, consisted of concentric layers including markedly hypointense in central tumor regions and peripheral zones which were moderately hypointense relative to liver parenchyma (arrows). **b** Coalescent lesion with target sign on fat-saturated T2-weighted TSE image, consisted of concentric layers including markedly hyperintense in central tumor regions and moderately hyperintense peripheral zone (arrows). **c** Lollipop sign on portal phase image, consisted of a well-defined tumor (as the “candy”) and an obstructed hepatic vein (as the “stick”) (arrow)

**Fig. 2:**
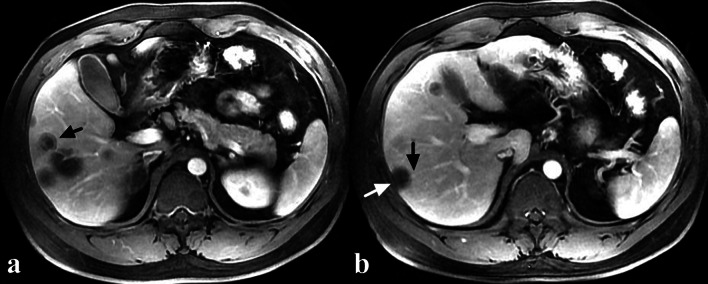
A 35-year-old male HEH patient. **a** Target sign on portal phase image, consisted of concentric layers including markedly hypointense in central tumor regions and peripheral zones which were moderately hypointense relative to liver parenchyma (arrow). **b** Capsular retraction caused by a subcapsular lesion (white arrow) and lollipop sign, consisted of a well-defined tumor (as the “candy”) and an obstructed portal vein (as the “stick”) (black arrow) on portal phase image

A total of 412 lesions were evaluated, including 108 in the left liver, 291 in the right liver, 12 in the middle (lesions locate in the middle part of liver and cannot be assigned to right of left lobe) and 1 in caudate lobe. Most of the lesions were nodular (80.3%), while coalescent and diffuse lesions accounted for 18.2% and 1.5%, respectively (Table 1) (Fig. 1a, b). One hundred and sixty-four lesions (39.8%) were subcapsular and 47 of them (11.4%) had the sign of capsular retraction (Fig. 2b). On T1W images, target sign only appeared in 65 lesions (15.8%), while low SI (84.2%) was more commonly observed (Fig. 1a). On T2W images, target sign appeared in 196 lesions (47.6%) and high SI accounted for 52.4% (Fig. 1b). Three hundred and sixty lesions (87.4%) showed high SI on DWI images and target sign appeared in 52 lesions (12.6%). On dynamic contrast enhanced T1W images, ring-like enhancement was the most pattern in arterial phase (49.5%), while target-like enhancement was more common both in portal phase (35.4%) and equilibrium phase (34.0%) (Table1) (Fig. 2a). Lollipop sign was observed in 60 lesions (14.6%) (Fig. 1c, 2b).

**Table 1:** MRI characteristics of all evaluated intrahepatic lesions of HEH patients

Parameters	Number of lesions (*n* = 412)	Ratio (%)	Parameters	Number of lesions (*n* = 412)	Ratio (%)
*Location*			*DWI*		
Left	108	26.2	High signal intensity	360	87.4
Right	291	70.6	Target	52	12.6
Middle	12	3.0			
Caudate lobe	1	0.2	*Enhanced pattern*		
*Lesion type*			Arterial		
Nodular	331	80.3	None	117	28.4
Coalescent	75	18.2	Ring	204	49.5
Diffuse	6	1.5	Target	45	10.9
*Subcapsular lesion*			Core	13	3.2
Yes	164	39.8	Heterogeneous	33	8.0
No	248	60.2	Portal		
*Capsular retraction*			None	45	10.9
Yes	47	11.4	Ring	133	32.3
No	365	88.6	Target	146	35.4
*Lollipop sign*			Core	21	5.1
Yes	60	14.6	Heterogeneous	67	16.3
No	352	85.4	Equilibrium		
*T1W*			None	45	10.9
Low signal intensity	347	84.2	Ring	133	32.3
Target	65	15.8	Target	140	34.0
*T2W*			Core	21	5.1
High signal intensity	216	52.4	Heterogeneous	73	17.7
Target	196	47.6

### Comparison of MRI features among HEH lesions of different sizes

**Fig. 3:**
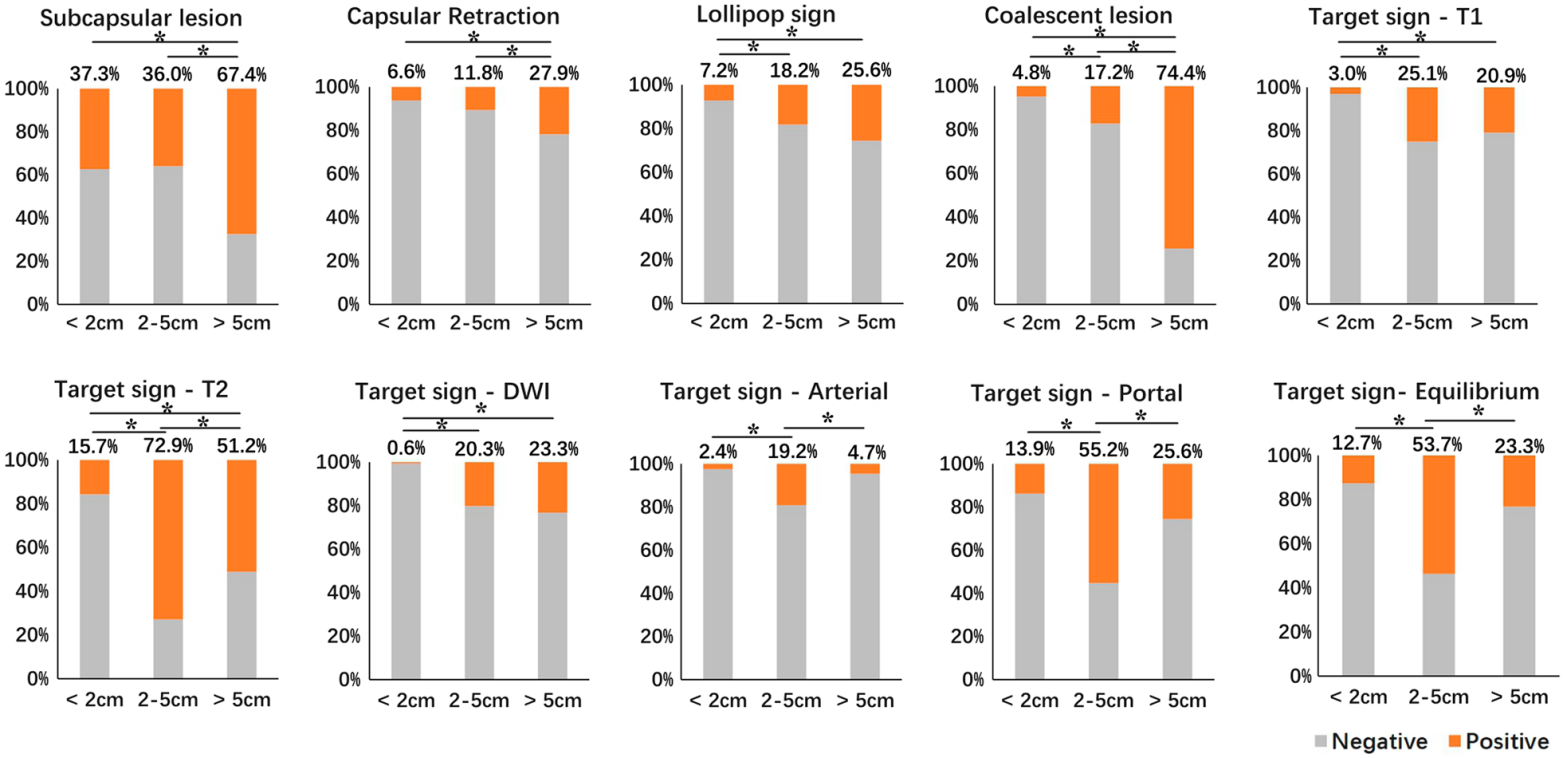
The comparison results of different MRI features among lesions of different sizes

Based on the size, the evaluated lesions were categorized into three groups: small (size < 2 cm, *n* = 166, 40.3%), medium (size 2–5 cm, *n* = 203, 49.3%) and large (size > 5 cm, *n* = 43, 10.4%) and MRI features were compared among them (Table 2). Compared with small and medium group, the large group had significantly higher rate of subcapsular lesion and capsular retraction, (*p* < 0.01, respectively) (Fig. 3). As the lesion size grew, the rate of lollipop sign and coalescent lesion got higher and significant differences were detected in the comparison of small versus medium (*p* < 0.01) and small versus large (*p* < 0.01). The difference in the rate of coalescent lesion between medium and large was also significant (*p* < 0.01).

**Table 2:** Comparison of MRI features among lesions in different size groups

Sign	Size group			*p* value
	Small (< 2 cm) (*n* = 166)	Medium (2–5 cm) (*n* = 203)	Large (> 5 cm) (*n* = 43)	
Subcapsular lesion	62 (37.3%)	73 (36.0%)	29 (67.4%)	< 0.01
Capsular retraction	11 (6.6%)	24 (11.8%)	12 (27.9%)	< 0.01
Lollipop sign	12 (7.2%)	37 (18.2%)	11 (25.6%)	< 0.01
Coalescent lesion	8 (4.8%)	35 (17.2%)	32 (74.4%)	< 0.01
*Target sign*				
T1W	5 (3.0%)	51 (25.1%)	9 (20.9%)	< 0.01
T2W	26 (15.7%)	148 (72.9%)	22 (51.2%)	< 0.01
DWI	1 (0.6%)	41 (20.3%)	10 (23.3%)	< 0.01
Arterial	4 (2.4%)	39 (19.2%)	2 (4.7%)	< 0.01
Portal	23 (13.9%)	112 (55.2%)	11 (25.6%)	< 0.01
Equilibrium	21 (12.7%)	109 (53.7%)	10 (23.3%)	< 0.01

**Fig. 4:**
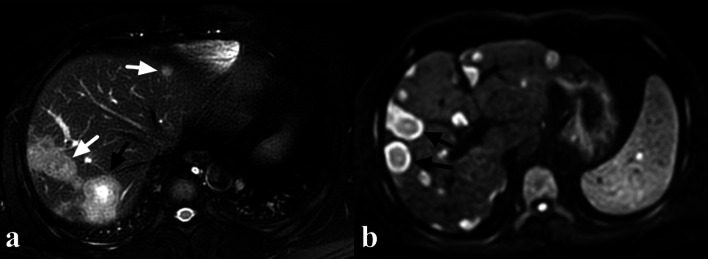
A 38-year-old male HEH patient. **a** On fat-saturated T2 weighted image, target sign could be observed on a medium lesion (black arrow), while both small and large lesions failed to show target appearance (white arrow). **b** Target sign on DWI image (800 b-value), consisted of concentric layers including relatively hyperintense in central tumor regions and peripheral zones which were markedly hyperintense relative to liver parenchyma (arrows)

**Fig. 5:**
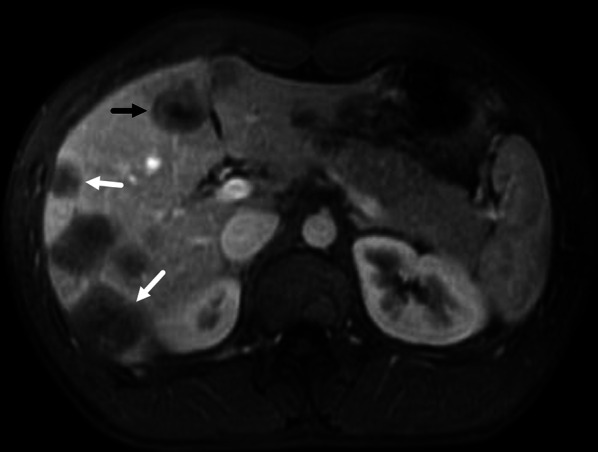
A 22-year-old male HEH patient. On portal phase image, target sign could be observed on a medium lesion (black arrow), while both small and large lesions failed to show target appearance (white arrow)

Medium group had the highest rate of target sign on both T2W (72.9%) and T1W (25.1%) images, and the differences were significant in comparison with small versus medium and small versus large on both sequences (*p* < 0.01, respectively) (Fig. 3, 4a). The difference in the rate of target sign on T2W images between medium and large was also significant (*p* < 0.01). Both large and medium groups had higher rate of target sign on DWI images, compared with small group (both *p* < 0.01) (Fig. 4b). Medium group had the highest rate of target sign on arterial phase (19.2%), portal phase (55.2%) and equilibrium phase (53.7%) images, and the differences were significant in comparison with medium versus small and medium versus large on these sequences (*p* < 0.01, respectively) (Figs. 3, 5).

### Comparison of MRI features between HEH and HMT on per-patient analysis

**Fig. 6:**
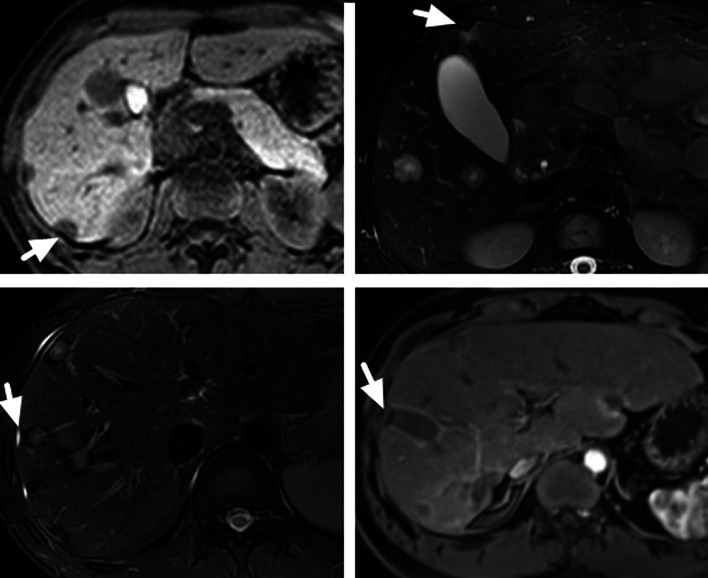
The appearance of capsular retraction on MRI images of 4 HEH patients (arrows)

**Fig. 7:**
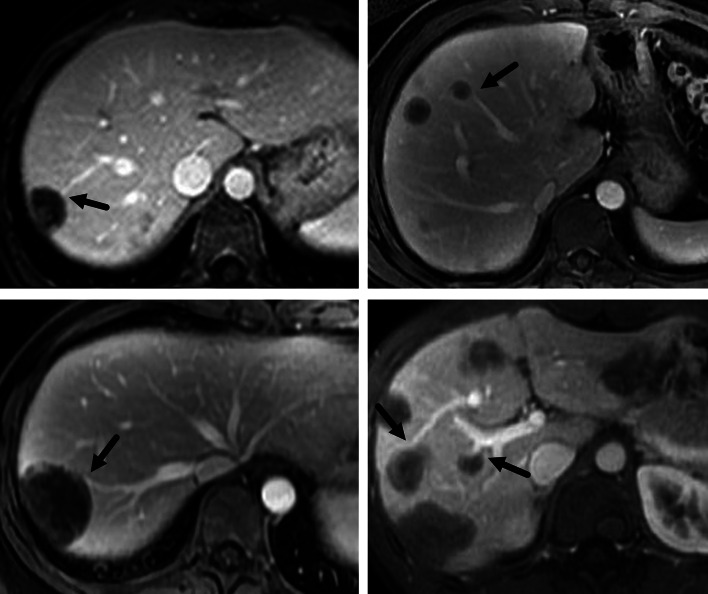
The appearance of lollipop sign on MRI images of 4 HEH patients (arrows)

**Fig. 8:**
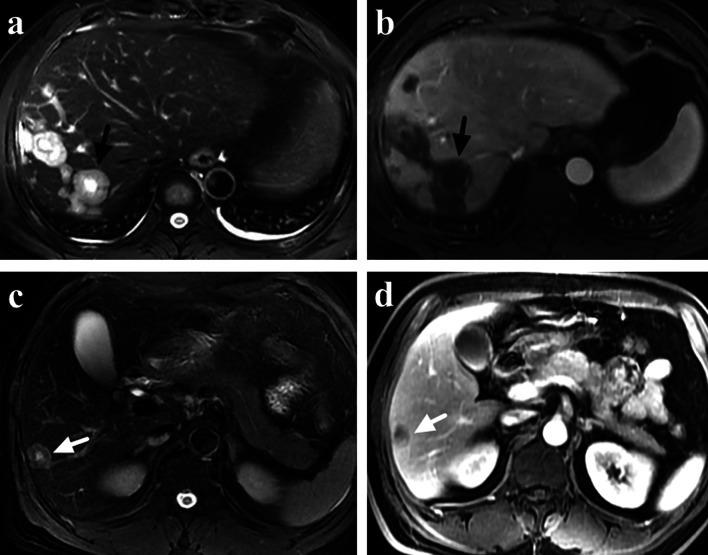
**a, b** A 40-year-old male HEH patient with coexistence of target appearance on both T2 weighted and portal phase images. **a** Target sign on fat-saturated T2-weighted image, consisted of concentric layers including markedly hyperintense in central tumor regions and moderately hyperintense peripheral zone (arrow). **b** Target sign on portal phase image, consisted of concentric layers including markedly hypointense in central tumor regions and peripheral zones which were moderately hypointense relative to liver parenchyma (arrow). **c, d** A 51-year-old male rectal carcinoma patients with multiple liver metastasis. **c** A classic target sign on fat-saturated T2-weighted image (arrow). **d** Heterogeneous enhancement on portal phase image (arrow), instead of target sign

From September 2020 to September 2021, a total of 836 patients had abdominal contrast-enhanced liver MRI in our center and 102 patients were radiologically diagnosed as HMT. Finally, 63 HMT patients with histological results and pre-treatment contrast-enhanced liver MRI were included in this study. The primary tumor sites included colorectum (24, 38.1%), pancreas (19, 30.2%), bile duct (9, 14.3%), breast (6, 9.5%) and stomach (5, 7.9%). No difference was found between HEH and HMT patients in gender and the number of lesions, while HEH patients were much younger than HMT patients (*p* < 0.01) (Table 3). There were significant differences between HEH and HMT patients in the features of subcapsular lesion, capsular retraction, coalescent lesion and lollipop sign (*p* < 0.01, respectively). Compare with HEH patients, HMT patients seldom had the appearance of lollipop sign (66.7% versus 6.4%) and capsular retraction (59.6% versus 3.2%) (Table 3) (Figs. 6, 7). Target sign was more commonly found on T2W and portal phase images in both HEH (75.4% and 70.2%) and HMT (39.7% and 36.5%) patients. There were significant differences between HEH and HMT patients in the feature of target sign on MRI images of T1W, T2W, portal phase and equilibrium phase (*p* < 0.01, respectively), while no difference was found on DWI and arterial phase images (Table 3). If target sign was defined as positive as lesions presented target appearance on any sequence, no difference was found between HEH and HMT patients (*p* = 0.089). If target sign was defined as positive as lesions presented target appearance on both T2W and portal phase, HEH patients had significantly higher positive rate compared with HMT patients (64.9% versus 12.7%, *p* < 0.01) (Fig. 8).

**Table 3:** Comparison of MRI features between HEH and HMT patients

Parameters	HEH (*n* = 57)	HMT (*n* = 63)	*p* value
*Gender*			
Male	32 (56.1%)	38 (60.3%)	*p* = 0.643
Female	25 (43.9%)	25 (39.7%)	
*Age (years)*	37.3 ± 11.7	60.8 ± 10.8	*p* < 0.01
*Number of lesions*			
Singular	5 (8.8%)	11 (17.5%)	
2–10	28 (49.1%)	35 (55.5%)	*p* = 0.140
> 10	24 (42.1%)	17 (27.0%)	
*MRI features*			
Subcapsular lesion	51 (89.5%)	31 (49.2%)	*p* < 0.01
Capsular retraction	34 (59.6%)	2 (3.2%)	*p* < 0.01
Lollipop sign	38 (66.7%)	4 (6.4%)	*p* < 0.01
Coalescent lesion	33 (57.9%)	19 (30.2%)	*p* < 0.01
Target sign			
T1W	25 (43.9%)	11 (17.5%)	*p* < 0.01
T2W	43 (75.4%)	25 (39.7%)	*p* < 0.01
DWI	18 (31.6%)	20 (31.7%)	*p* = 0.571
Arterial	17 (29.8%)	18 (28.6%)	*p* = 0.519
Portal	40 (70.2%)	23 (36.5%)	*p* < 0.01
Equilibrium	38 (66.7%)	21 (33.3%)	*p* < 0.01
Any sequence	46 (80.7%)	43 (68.3%)	*p* = 0.089
T2W + Portal	37 (64.9%)	8 (12.7%)	*p* < 0.01

## Discussion

HEH is an extremely rare disease and the discrepancy of biological behavior could lead to huge differences in the long-term survival [5, 11]. For most patients, HEH were detected occasionally by ultrasound or CT with no symptom and the progression of the tumor was slow [4]. Moreover, spontaneously tumor regression was reported in some HEH patients [3, 5, 6]. Liver transplantation and surgical resection have been reported with good long-term results [12–18]. However, for most of HEH patients, intrahepatic lesions were evaluated as unresectable, since multiple lesions were observed in both lobes of the liver. Even for HEH patients with singular intrahepatic lesion, extrahepatic metastasis (lung, bone, spleen, etc.) may coexist, which made radical resection impossible. Due to the nearly normal liver function of HEH patients and limited organ donation, liver transplantation may be unaccessible for most HEH patients. Recently, sirolimus and interferon-a 2b have been reported with the satisfying results, which makes them good treatment options [19–23]. Although the MRI characteristics of HEH have been reported, radiological misdiagnosis of HEH was very common and metastatic tumor was the most scenario, which made it a necessity to summarize the features of MRI appearances in a large group of HEH patients. In this study, the MRI images of 57 HEH patients were retrospectively analyzed on both per-lesion and per-patient evaluation. From our knowledge, this study included the most HEH patients for MRI features analysis ever reported, which made the results much more comprehensive and reliable.

In this study, the characteristic MRI findings of HEH were previously reported in the literatures, such as coalescent lesion, subcapsular lesion, capsular retraction, lollipop sign and target sign [7–9, 24–26]. According to the results of this study, all of these features were related to the lesion size and small lesions (< 2 cm) had the lowest rates in all of them, except for subcapsular lesion. Although coalescent lesion was reported to be one characteristic of HEH, most of the lesions were found to be nodular in this study and coalescent lesions only accounted for a small proportion. The occurrence of coalescent lesion correlated with the size. As the lesion grew, the possibility of a merged lesion from several nodular lesion increased. In this study, rate of coalescent lesion was 74.4% in the group of large lesions (> 5 cm). Compared with HMT, HEH patients had a higher rate of coalescent lesion, but 30.2% HMT patients still had the appearance of coalescent lesion, which made it a less specific feature of HEH. The same situation applied to subcapsular lesion. Subcapsular lesion was also deemed as a feature of HEH [27, 28], but almost half of HMT patients had the appearance of subcapsular lesion, which could not be used as a feature for differential diagnosis.

In this study, a total of 47 lesions (11.4%) caused capsular retraction and 34 patients (59.6%) had this appearance, which was in agreement with previous literatures [6, 8, 29]. The possibility of capsular retraction also correlated with the size of lesion. The lesion in the large group (> 5 cm) had the highest rate of capsular retraction. Meanwhile, HMT patients seldom had the appearance of capsular retraction which made it a specific radiological feature for HEH. Lollipop sign which was depicted as a combination of two structures: the well-defined tumor on contrast enhanced images (as the “candy”) and the occluded vein (as the “stick”), was previously reported to be a characteristic sign of HEH [30, 31]. In this study, we found that 14.6% of lesions had the appearance of lollipop sign and its occurrence also correlated with the size of lesion. The lesions in large group (> 5 cm) had the highest rate of lollipop sign. Moreover, lollipop sign was found in 66.4% of HEH patients, while HMT patients rarely had this appearance, which made it another specific feature for differential diagnosis.

Target sign which was depicted as a stratified pattern with concentric rings of varying intensity was reported to be a specific MRI feature for HEH [32–35]. It was generated by a sclerotic fibrous center, a layer of cellular proliferation and a peripheral narrow avascular zone between the tumor and liver parenchyma. Although target sign could be found on different MRI sequence images, T2W and portal phase had higher rate of target sign [36]. In this study, on per-lesion analysis, T2W had the highest rate of target sign. The occurrence of target sign also correlated with the size lesion and the medium group (2–5 cm) had the highest rate. For small lesions (< 2 cm), a sclerotic fibrous center may be lacked and large lesions (> 5 cm) may have irregular shape of sclerotic center, instead of a round shape. Although a difference could be detected between HMT and HEH patients in the rate of target sign on T1W, T2W, portal and equilibrium phase images, more than half of HMT patients had target sign on T2W images, which made it a less specific feature for HEH. However, if the target sign was defined as positive in the setting of target appearance on both T2W and portal phase, the rate of target sign in HMT decreased obviously. So, coexistence of target appearance on both T2W and portal phase could be a more specific MRI feature for HEH, instead of target sign on only one sequence.

There were several limitations about this study. First, HMT from different primary tumors may impact the viability to distinguish HEH from each of them, especially for cholangiocarcinoma, due to the possible overlapping characteristic of capsular retraction. However, for patients with HMT, symptoms, abnormalities of laboratory tests and radiological findings caused by primary tumors will also be helpful for differential diagnosis. The purpose of this study was to provide more accurate depiction of HEH MRI features, based on a larger patients group. But from our point of view, MRI appearances of HEH could differ obviously among patients and the intra-patient heterogeneity should also be noticed. The value of depicted MRI features in this study was to provide radiological clues and the final diagnosis of HEH should still rely on pathological examination. Second, MRI features on hepatobiliary phase were previously reported to be valuable for HEH [36]. Since hepatobiliary phase was not performed for most HEH patients in this study, the characteristics of hepatobiliary phase was not analyzed.

In conclusion, capsular retraction and lollipop sign were specific features of HEH and larger lesions (> 5 cm) had higher possibility to present these appearances. Since both of them were rarely presented in HMT patients, capsular retraction and lollipop sign could be used for quick distinguishment of HEH. Medium lesions (2–5 cm) of HEH were more likely to present target sign. Because target sign on T2W or portal phase was not rare in HMT patients, it may not be suitable for differential diagnosis. However, the coexistence of target appearance on both T2W and portal phase images was relatively rare in HMT patients, which made it a specific MRI feature for HEH.

## Data Availability

The data used during this study are available from the corresponding author on reasonable request.

## Abbreviations

CT: Computed tomography
DWI: Diffusion-weighted imaging
GRE: Gradient recall echo
HEH: Hepatic epithelioid hemangioendothelioma
HMT: Hepatic metastatic tumors
MRI: Magnetic resonance imaging
SI: Signal intensity
TSE: Turbo spin echo
W: Weighted
